# Supplementary material for: "Even though I am alone, I feel that we are many" - An appreciative inquiry study of asynchronous, provider-to-provider teleconsultations in Turkana, Kenya
Source: PLoS One. 2020 Sep 15;15(9):e0238806. doi: 10.1371/journal.pone.0238806 (PMC7491713; doi:10.1371/journal.pone.0238806)
Supplement: Attachment — Submitted filename: Response to reviewers.pdf [file pone.0238806.s002.pdf]

Response to review comments

Manuscript ID: PONE-D-20-00735

Manuscript title: "Even though I am alone, I feel that we are many" – An appreciative inquiry study of asynchronous, provider-to-provider

	Comment	Response
**Journal**		
1	Please ensure that your manuscript meets PLOS ONE's style requirements, including those for file naming. The PLOS ONE style templates can be found at http://www.plosone.org/attachments/PLOSOne_formatting_sample_main_body.pdf and http://www.plosone.org/attachments/PLOSOne_formatting_sample_title_authors_affiliations.pdf	We have renamed files and made changes according to the style requirements.
2	Please include a copy of the interview guide used in the study, in both the original language and English, as Supporting Information, or include a citation if it has been published previously.	The key informant interview and focus group guides have been uploaded to the Qualitative Data Repository: https://doi.org/10.5064/F6UURYON<br>Links to the repository have been included in the manuscript.
3	Thank you for providing the following Funding Statement: "The work and all authors were supported by a County Innovation Challenge Fund award from DFID (CICF-INN-R1-033) to Health-E-Net Limited. PK was also supported by a Stars in Global Health award (S5 0420-01) from Grand Challenges Canada. The funders had no role in study design, data collection and analysis, decision to publish, or preparation of the manuscript." We note that one or more of the authors is affiliated with the funding organization, indicating the funder may have had some role in the design, data collection, analysis or preparation of your manuscript for publication; in other words, the funder played an indirect role through the participation of the co-authors. If the funding organization did not play a role in the study design, data collection and analysis, decision to publish, or preparation of the manuscript and only provided financial support in the form of authors' salaries and/or research materials, please review your statements relating to the author contributions, and ensure you have specifically and accurately indicated the role(s) that these authors had in your study in the Author Contributions section of the online submission form. Please make any necessary amendments directly within this section of the online submission form. Please also update your Funding Statement to include the following statement: "The funder provided support in the form of salaries for authors [insert relevant initials], but did not have any additional role in the study design, data collection and analysis, decision to publish, or preparation of the manuscript. The specific roles of these authors are articulated in the 'author contributions' section."	Funding was provided from DFID through the County Innovation Challenge Fund managed by Options Consulting Services. None of the authors were or are affiliated with the funding organisation or its affiliates. Neither the the funding organisation nor its affiliates played any role in the study design, data collection and analysis, decision to publish, or preparation of the manuscript. The funding statement has been amended to include the following: "The funder provided support in the form of salaries for authors SS, AM, VK and PK, but did not have any additional role in the study design, data collection and analysis, decision to publish, or preparation of the manuscript. The specific roles of these authors are articulated in the 'author contributions' section."

	Comment	Response
4	We note that you have stated that you will provide repository information for your data at acceptance. Should your manuscript be accepted for publication, we will hold it until you provide the relevant accession numbers or DOIs necessary to access your data. If you wish to make changes to your Data Availability statement, please describe these changes in your cover letter and we will update your Data Availability statement to reflect the information you provide.	The research codebook with excerpts, along with data collection consent forms and discussion guides, have been uploaded to QDR and can be accessed with the following DOI number: https://doi.org/10.5064/F6UURYON. Due to ethical considerations and informed consent agreements with research participants, we are unable to upload raw data (transcripts or respondent drawings).
**Reviewer 1**		
1	This is a qualitative study using Appreciative Inquiry that studied the impact of asynchronous tele-consultations on providers and health systems in Kenya. Overall, this is a very lengthy, verbose manuscript. The authors should critically review to make the content more concise.	We have reviewed the entire manuscript in detail to make it more concise and readable. This inlucdes moving some sections into two tables: one for respondent types and another to summarise/highlight respondent responses along the different stakeholder groups identified. Some sections have been removed (e.g. discussion on voluntarism, which will be explored in a separate manuscript) while others have been rephrased to emphasise readability.
2	Abstract: This may read better if the authors use a more active voice, e.g. "We demonstrated that provider-to-provider asynchronous tele-consultations impacted multiple stakeholders."	The abstract has been changed to reflect this comment.
3	The last portion of the final sentence is inaccurate as the authors did not study "patient-level health outcomes". Please rephrase to be more accurate, e.g. "...health service delivery interventions that can benefit providers and health systems."	The abstract has been changed to reflect this comment.
4	Introduction: The third sentence is lengthy and difficult to understand. Please rephrase.	This has been simplified by splitting into two sentences to improve clarity.
5	I would recommend that the authors do a more extensive literature as some of the background is inaccurate. This includes the final sentence of the first paragraph. For example, many studies have been published on telestroke that demonstrate both its clinical and cost effectiveness. Similarly, there are publications on asynchronous telemedicine services beyond tele-dermatology, i.e. tele-radiology, tele-ophthalmology, etc.	In the literature, we found many examples of pilots and small-scale projects that showed some evidence under controlled conditions. However, our intention here was to emphasize the lack of evidence at scale. We have added the words 'at scale' to reflect this. We also qualify ths sentence to focus on LMICs, which is the main intent of this publication.
6	At the end of the introduction, the authors include three objectives. It does not seem that the third objective was addressed in the body of the manuscript, i.e. how ATCs can be sustained in LMIC health systems. Please remove this objective.	We agree as indeed, we this manuscript focuses on the "Discovery Phase" of Appreciative Inquiry which explores "what worked" and this was clearly highlighted. The sentence was revised to emphasize the potential influence of this research on future health systems.

	Comment	Response
7	Materials and Methods: The authors state that research participants were recruited based on frequency of usage of the nREM system. This is concerning as it could introduce significant selection bias into the study. That is, by only collecting data from people who frequently used the system, they are missing data from those who did not use it. This is a major limitation as the non-users may have a very different experience with and impression of the impact of nREM on providers, patients, and the healthcare system.	Information on research participants and recruitment has been moved to the Table 1. This clarifies that the recruitment was based on having conducted at least two ATCs. This ensured that research participants (especially NPCs) had experience with the system, but did not bias against those with infrequent use. Frequency of use applied only to remote specialists and not to NPCs and MOs in Turkana. We don't believe this biases the study, as remote physicians were engaged based on clinical need (and not familiarity with the system), and their comments were linked to the cases rather than the technical aspects of using the system.
8	Results: Under the section "Improved Skills and Confidence", how did this system improve the technical skills if it was asynchronous? This is not intuitive. Please provide an example. It is much easier to understand how the asynchronous consults would improve knowledge and confidence -- but technical skills seems less likely.	Examples highlighting this have been provided in Table 2. The text includes the following quote by an NPC: “I feel satisfied because when I enter the case, I get more knowledge because the doctors or the consultants will add more flesh to what I have, to my diagnosis, when they now send the feedback. I am able to see it and say, ‘Ok, I could have done this, I could have done that.’ So, it adds some more knowledge to me in my experience”. We have highlighted that improvement in skills and confidence are not restricted to real-time telemedicine.
9	For the quantitative data on referrals vs tele-consultations (Figure 4), please do a comparative analysis to determine whether the difference is statistically significant.	The difference between groups was not significant (X2 (4, N = 1,078) = 7.1703, p = .13. We have included this in the figure caption and manuscript. Despite the lack of significance, it is important to highlight areas where differences could be seen in future implementations (with larger numbers).
10	Under "Benefits to Patients and Care givers", the first sentence states that there is a "direct association with patient-level outcomes...." This is inaccurate as no patient-level outcomes are reported in this study. I would rephase to something like "patient level experience".	This sentence now begins with "As described by both NPCs and remote physicians ..." to highlight that the effect on patients/care-givers was inferred from healthcare providers.
11	Discussion: In the second paragraph on Page 26, the authors should rephrase the first sentence. There were no intermediate patient-level outcomes measured. It is more accurate to say that this is the NPCs perception of the patient experience as there was no data collected directly from the patients, e.g. via surveys, interviews, focus groups, etc.	This sentence includes the phrace "as perceived by NPC respondents upon reflection on patient experience"
12	Please remove "Akin to dating apps" in the conclusion. This has no place in medical literature.	This has been removed

	Comment	Response
**Reviewer 2**		
1	Reviewer #2: Comments also attached. I think this is a nice study, and adds a value to in terms of provider engagement and addressing access to health care in low recourse communities. It would be great to revise the way it's written. It was a little bit hard to read at some points. I also would comment on the sustainability of the platform	The authors have discussed potential future implications of ATCs on LMIC health systems, as drawn from the results of this study. Aspects of sustainability are not addressed here, as we have focused this study on impact of ATCs specifically (as also pointed out by the first reviewer), without addressing the specific sustainability elements of the nREM platform. Future studies will address this.
2	Introduction section: Standardize the terminology used: Doctor to doctor vs provider to provider	The authors have strategically chosen "provider" in this manuscript as to include the range of medically trained personnel in this context. The interaction occuring on the nREM platform, and what we hope to convey in ATCs generally, is not strictly between doctors.<br>We have reviewed the manuscript for consistency.
3	It might be beneficial to describe the scope of practice of the NPC.	We have included a reference and the following sentence in the introduction: While the scope of practice of each cadre of NPC varies by country, the number of NPCs have been steadily increasing as LMICs address the need for additional human resources to deliver healthcare services."
4	Page 9 Under MATERIALS AND METHODS section: The way it written, it is describing Appreciative Inquiry methodology study design rather than the valuable work that the study intends to deliver. It would be great to describe the demographics of not only the subjects but the type of the consults, include/move the details of platform, training of the providers and measurements in this section. This will help the reader to understand the value of “understanding how the asynchronous tele-consultation intervention contributed to social and systems-level advances, and how these improvements can be leveraged to integrate tele-consultations”	We thank the reviewer for highlighting the importance of this work, and we have made the following changes:<br>1. Moved the description of the intervention into the methods section<br>2. Provided more information on the use and case mix in the beginning of the results serction.<br>The focus of the study was to highlight the diverse impacts of ACTs, and the authors believe that the Appreciative Inquiry methodology was best suited to convey these findings. Future studies are planned to document the specific elements of this platform that have been recommended, as these benefits deserve increased attention.
5	Abbreviations should be explained within parentheses at their first mention in the manuscript.	This has been reviewed and addressed

	Comment	Response
6	Page 10-11: Would like to see a description of the data included	Interview/FDG guides and the analysis code book have been provided in the Qualitative Data Repository: https://doi.org/10.5064/F6UURYON The methods section also has been revised to include information on the data collected in the study.
7	Facilitation of key informant interviews (KIIs) and focus group discussions (FGDs) was conducted by a public health and Appreciative Inquiry specialists within the Health-E-Net team: Few suggestions/comments: Unclear if the was a standardized interview tool utilized	The study team did apply a standardized interview tool for both KIIs and FGDs. The text has been amended to explain where these guides can be located (QDR).
8	Is there any professional relationship between the team and the interviews (volunteer consulting physicians (MOs and specialists) ?? if so, what other measures used to minimize biases?	The relationship between the interviewer and respondents was a professional one as researcher conducting a study; we have included in the limitations a note about potential social desirability bias.
9	Page 12-Under the results questions: It seems that there were some open-ended questions and story telling as mentioned in the limitation section, it might be more beneficial to have standardized questions to address the feasibility, challenges and benefits. Information may be difficult to quantify or organize	Data were collected following the first three phases of Appreciative Inquiry (Discovery, Dream, and Design), while only data from the first phase were analyzed and documented in this manuscript. Data collection did follow a semi-structured interview guide, now referenced in the text and submitted to QDR for access.
10	Page 20-21: It might be more powerful to describe the impact of ATC on patient care e.g description of avoided transfers, number of patients who were able to receive their care at their home town	This study aims to highlight the diverse impacts of ACTs, and the authors believe that the Appreciative Inquiry methodology was best suited to convey these findings. Future studies are planned to document the specific elements of this platform that have been recommended, as these benefits deserve increased attention.
