# Supplementary material for: "Even though I am alone, I feel that we are many" - An appreciative inquiry study of asynchronous, provider-to-provider teleconsultations in Turkana, Kenya
Source: PLoS One. 2020 Sep 15;15(9):e0238806. doi: 10.1371/journal.pone.0238806 (PMC7491713; doi:10.1371/journal.pone.0238806)
Supplement: Attachment — Submitted filename: Response to reviewers-20200810.pdf [file pone.0238806.s003.pdf]

**Response to review comments - 2020-07-10**

Manuscript ID: PONE-D-20-00735

Manuscript title: “Even though I am alone, I feel that we are many” – An appreciative inquiry study of asynchronous, provider-to-provider teleconsultations in

| | Comment | Response |
|---|---|---|
| **Reviewer #3** | | |
| 1 | on the Title, Author, Affiliation page, the authors need to revisit the Plos One formatting sample and make corrections accordingly. | The title page has been reformatted |
| 2 | Reviewer 1, comment 5 suggests the authors need a more extensive literature review incorporating newer (or other) articles reporting on asynchronous telemedicine services. While it is acceptable to argue that the focus of the current article is on studies "at scale" the existence of a broader literature can still be acknowledged. I would suggest something along the lines of "while there is a growing literature on the use of asynchronous telemedicine applications to assist medical providers in underserved areas (provide several references here), the need for studies on applications at scale remains." Or something like this. In this way you can acknowledge there are studies on asynchronous applications and still justify the importance of your study by highlighting how it differs from these. | We have added a sentence to the second paragraph of the introduction and listed a number of references to studies/implementations of asynchronous telemedicine in underserved areas. The addition acknowledges the other efforts, and the need for further study into the specfic telemedicine paradigm. |
| 3 | Under Materials and Methods, after Figure 1 paragraph, the paragraph beginning "a volunteer network..." Sentence 2 reads "The network included current and former employees the county..." In your edits you removed too much here. Should read "...current and former employees of the county..." | Thanks for spotting this. We have made the correction. |
