# Supplementary material for: "Even though I am alone, I feel that we are many" - An appreciative inquiry study of asynchronous, provider-to-provider teleconsultations in Turkana, Kenya
Source: PLoS One. 2020 Sep 15;15(9):e0238806. doi: 10.1371/journal.pone.0238806 (PMC7491713; doi:10.1371/journal.pone.0238806)
Supplement: Attachment — Submitted filename: PLOS.docx [file pone.0238806.s001.docx]

Dear Drs Fry, Saidi, Musa, Kithyoma and Kumar

Thank you for your work to ensure access to your patients regardless of their location.

Nice and valuable work to demonstrate the value of provider engagement in low-middle income communities to deliver health care by connecting rural providers and their patients to services at distant sites and promoting patient-centered health care and equal access

Introduction section:

Standardize the terminology used:

Doctor to doctor vs provider to provider

It might be beneficial to describe the scope of practice of the NPC.

Page 9

Under MATERIALS AND METHODS section:

The way it written, it is describing Appreciative Inquiry methodology study design rather than the valuable work that the study intends to deliver. It would be great to describe the demographics of not only the subjects but the type of the consults, include/move the details of platform, training of the providers and measurements in this section. This will help the reader to understand the value of “understanding how the asynchronous tele-consultation intervention contributed to social and systems-level advances, and how these improvements can be leveraged to integrate tele-consultations”

Abbreviations should be explained within parentheses at their first mention in the manuscript.

Page 10-11:

Would like to see a description of the data included

**Facilitation of key informant interviews (KIIs)** and focus group discussions (FGDs) was

conducted by a public health and Appreciative Inquiry specialists within the Health-E-Net

team:

Few suggestions/comments:

- Unclear if the was a standardized interview tool utilized
- Is there any professional relationship between the team and the interviews (volunteer consulting physicians (MOs and specialists) ?? if so, what other measures used to minimize biases?

Page 12-

Under the results questions:

- It seems that there were some open-ended questions and story telling as mentioned in the limitation section, it might be more beneficial to have standardized questions to address the feasibility, challenges and benefits
- Information may be difficult to quantify or organize

Page 20-21

It might be more powerful to describe the impact of ATC on patient care e.g description of avoided transfers, number of patients who were able to receive their care at their home town
